# Buprenorphine-elicited alteration of adenylate cyclase activity in human embryonic kidney 293 cells coexpressing κ-, μ-opioid and nociceptin receptors

**DOI:** 10.1111/jcmm.12644

**Published:** 2015-07-08

**Authors:** Pei-Chen Wang, Ing-Kang Ho, Cynthia Wei-Sheng Lee

**Affiliations:** a Neuropsychiatric Center, National Health Research InstitutesMiaoli County, Taiwan; b Graduate Institute of Clinical Medical Science, China Medical UniversityTaichung, Taiwan; c Center for Drug Abuse and Addiction, China Medical University HospitalTaichung, Taiwan; d China Medical UniversityTaichung, Taiwan

**Keywords:** adenylate cyclase activity, buprenorphine, opioid receptors

## Abstract

Buprenorphine, a maintenance drug for heroin addicts, exerts its pharmacological function *via* κ- (KOP), μ-opioid (MOP) and nociceptin/opioid receptor-like 1 (NOP) receptors. Previously, we investigated its effects in an *in vitro* model expressing human MOP and NOP receptors individually or simultaneously (MOP, NOP, and MOP+NOP) in human embryonic kidney 293 cells. Here, we expanded this cell model by expressing human KOP, MOP and NOP receptors individually or simultaneously (KOP, KOP+MOP, KOP+NOP and KOP+MOP+NOP). Radioligand binding with tritium-labelled diprenorphine confirmed the expression of KOP receptors. Immunoblotting and immunocytochemistry indicated that the expressed KOP, MOP and NOP receptors are *N*-linked glycoproteins and colocalized in cytoplasmic compartments. Acute application of the opioid receptor agonists— U-69593, DAMGO and nociceptin— inhibited adenylate cyclase (AC) activity in cells expressing KOP, MOP and NOP receptors respectively. Buprenorphine, when applied acutely, inhibited AC activity to ~90% in cells expressing KOP+MOP+NOP receptors. Chronic exposure to buprenorphine induced concentration-dependent AC superactivation in cells expressing KOP+NOP receptors, and the level of this superactivation was even higher in KOP+MOP+NOP-expressing cells. Our study demonstrated that MOP receptor could enhance AC regulation in the presence of coexpressed KOP and NOP receptors, and NOP receptor is essential for concentration-dependent AC superactivation elicited by chronic buprenorphine exposure.

## Introduction

Opioid addiction is a major public health problem that contributes to the significant morbidity and mortality related to HIV, hepatitis C and overdose 1,2. Opioids elicit their pharmacological responses through opioid receptors. Three conventional opioid receptors — μ (MOP), δ (DOP) and κ (KOP) — have been characterized 3–5. A non-opioid branch of opioid receptors has also been identified as opioid receptor-like 1 receptor 6 or the nociceptin/orphanin FQ peptide (NOP) receptor, with distinct pharmacology from those of conventional opioid receptors 7,8. The MOP, KOP and NOP receptors are colocalized in the cortex, amygdala, nucleus accumbens, dentate gyrus, superior colliculus, thalamus and hypothalamus in the central nervous system 9, suggesting that these three receptors might function cooperatively in neurons.

Buprenorphine is currently used in maintenance treatment programmes for heroin addicts 10,11. It has a long half-life and less abuse potential relative to other opioids 12, with ceiling effects in respiratory depression 13. Buprenorphine is a MOP receptor partial agonist and a potent KOP receptor antagonist 14 as well as a NOP receptor agonist 15,16. Despite being in clinical use for a long time, the molecular mechanism by which buprenorphine exerts its pharmacological effects remains unclear.

Adaptive changes in neurons underlie altered behaviours associated with opioid dependence and withdrawal syndrome 17. Prolonged exposure of NG108-15 neuroblastoma × glioma hybrid cells to morphine leads to increased adenylate cyclase (AC) activity 18, and this phenomenon may underlie the withdrawal state. Withdrawal of the agonist by adding the antagonist naloxone (precipitated withdrawal), which relieves the inhibition of AC by the agonist, revealed the phenomenon of AC superactivation or overshoot. Such regulation of AC could be a general means of cellular adaption to the alteration of opioid receptors 19,20.

The human embryonic kidney (HEK) 293 cell line is a widely distributed mammalian cell expression system and shares similar protein expression profiles with human neuronal cells 21. Previously, we demonstrated that methadone and buprenorphine exert initially different (acute exposure) but eventually convergent (chronic exposure) adaptive changes of AC activity in HEK 293 cells coexpressing human MOP and NOP receptors 22. Although the heterodimerized opioid receptors have been studied extensively 23–27, no cellular analysis of triply coexpressed KOP, MOP and NOP receptors after opioid exposure has been performed. In this study, we established an *in vitro* model overexpressing KOP, MOP and NOP receptors to investigate the pharmacodynamics of the coexpressed opioid receptors after buprenorphine treatment.

## Materials and methods

### Molecular cloning and expression in HEK 293 cells

Molecular cloning and stable expression of MOP and NOP receptors was performed previously 22. The N-terminal FLAG epitope (DYKDDDDA)-tagged human KOP receptor clone is a generous gift of Dr. Lee-Yuan Liu-Chen (Temple University School of Medicine, Philadelphia, PA, USA) 28. The cDNA encoding FLAG-tagged KOP receptor was subcloned into a mammalian expression vector, pCEP4 (Invitrogen, Carlsbad, CA, USA), which is a hygromycin-selectable vector. All sequences were verified by DNA sequence analysis.

Human embryonic kidney 293 cells harbouring human MOP or NOP receptors were grown in minimal essential medium (Invitrogen) supplemented with 10% foetal bovine serum, 100 units/ml penicillin, 100 μg/ml streptomycin, along with zeocin (0.5 mg/ml) or geneticin (0.5 mg/ml). Cell cultures were maintained at 37°C in a humidified 5% CO_2_ incubator 22. To stably express KOP receptor, the pCEP4 vector containing cDNA of FLAG-tagged human KOP receptor was transfected to HEK 293 cells by lipofection using FuGENE HD (F. Hoffmann-La Roche AG, Basel, Switzerland). Cell lines stably expressing FLAG-tagged human KOP receptor were selected by adding hygromycin (0.5 mg/ml) to the culture medium. Surface expression of FLAG-tagged human KOP receptor was confirmed by radioligand binding assays and measuring agonist-mediated inhibition of forskolin-induced cAMP accumulation.

### Receptor deglycosylation

Human embryonic kidney 293 cells stably expressing KOP, MOP or NOP receptors were grown to near confluence in 10-cm dishes. Cell extracts preparation, receptor deglycosylation by *N*-glycosidase F and immunoblotting were performed as described previously 22. For deglycosylation of KOP receptors, 20 μg of protein was first combined with Glycoprotein Denaturing Buffer (0.5% SDS, 40 mM DTT), and then incubated with 500 units PNGase F or Endo H (New England Biolabs, Ipswich, MA, USA) in G7 Reaction Buffer (50 mM sodium phosphate, pH 7.5 at 25°C) and 1% NP-40 at 37°C for 3 hrs. Membranes were incubated with polyclonal rabbit anti-DDDDK (equivalent to anti-FLAG; Abcam, Cambridge, UK), anti-KOP receptor (raised against the C-terminus of the human KOP receptor, DPAYLRDIDGMNKPV, and affinity-purified by GeneTex, Hsinchu, Taiwan), anti-MOP receptor (raised against the C-terminus of the human MOP receptor, TNHQLENLEAETAPLP, and affinity-purified by GeneTex), anti-NOP receptor (raised against the N-terminus of the human NOP receptor, MEPLFPAPFWEVIYGSHL and affinity-purified by ProSci Incorporated, Poway, CA, USA), monoclonal mouse anti-HA or anti-myc antibody (Cell Signaling Technology, Danvers, MA, USA) overnight at 4°C. After being washed, membranes were incubated with donkey anti-rabbit or sheep antimouse horseradish peroxidase-linked secondary antibody (GE Healthcare Life Sciences, Piscataway, NJ, USA). Immunoreactive proteins on the membrane were visualized by enhanced chemiluminescence (SuperSignal West Pico chemiluminescent substrate kit; Pierce Biotechnology, Rockford, IL, USA), and quantified using ImageQuant TL (GE Healthcare Life Sciences).

### Radioligand binding assays

The membrane preparation of HEK 293 cells were performed as described previously 22. The membrane pellet was resuspended in 50 mM Tris-Cl (pH 7.0) and 0.32 mM sucrose. Saturation radioligand binding assay was performed with opaque white 96-well filtre plates with FB glass fibre filtres (model MSFB N6B, Multiscreen Assay System; Millipore, Billerica, MA, USA). Cell membranes (7–8 μg of protein/well) were incubated with various concentrations of [15,16-^3^H]-diprenorphine (PerkinElmer Life Analytical Sciences, Boston, MA, USA) in binding buffer consisting of 50 mM Tris-Cl (pH 7.4), 1 mM EGTA (free acid), 10 μM leupeptin and 0.2% bovine serum albumin (BSA) for 1 hr at 25°C. Non-specific binding was determined by adding 3 μM naloxone (Tocris Bioscience, Bristol, UK) to the reaction mixture. The reaction was terminated by rapid filtration, and the filtres were washed three times with ice-cold binding buffer and dried at room temperature, overnight. After adding MicroScint-20 cocktail (PerkinElmer), bound radioactivity was measured using the TopCount NXT microplate scintillation and luminescence counter (PerkinElmer). Prism (GraphPad Software, La Jolla, CA, USA) was used to analyse the data derived from the saturation binding assay to obtain *B*_max_ and *K*_D_ values.

### Confocal microscopy and image analysis

Cells were grown on microscope cover glasses (Fisher Scientific, Pittsburgh, PA, USA) and incubated for 2 days prior to immunocytochemistry. After three washes in PBS^+^ (1× phosphate-buffered saline containing 1 mM MgCl_2_ and 0.1 mM CaCl_2_), cells were fixed with freshly prepared 4% paraformaldehyde in PBS^+^ for 30 min. at 4°C. After three washes with ice-cold PBS^+^ at 4°C, cells were permeabilized with 0.2% Triton X-100 in PBS^+^ at room temperature for 15 min. To remove excess Triton X-100, cells were washed five times with PBS^+^ at room temperature. Non-specific binding was then blocked by incubating the cells with 10% BSA in PBS^+^ at room temperature for 30 min. Immunostaining was performed by incubating the cells with 1:200 dilution of polyclonal rabbit anti-KOP receptor antibody (raised against the C-terminus of the human KOP receptor, DPAYLRDIDGMNKPV and affinity-purified by GeneTex), 1:50 dilution of monoclonal mouse anti-HA (Cell Signaling Technology), or 1:200 dilution of chicken anti-myc IgY fraction (Invitrogen) antibody in 10% BSA in PBS^+^ at 4°C overnight. The secondary antibody (1:500 dilution of Alexa Fluor 594 goat anti-rabbit, 1:200 dilution of Alexa Fluor 488 goat antimouse; Invitrogen, or 1:500 dilution of Alexa Fluor 647 donkey anti-chicken IgY antibody; Millipore) was applied for 1 hr at room temperature. Cells were then washed three times with PBS^+^ and mounted (ProLong Gold Antifade Kit; Invitrogen) for imaging. Images were acquired using a Leica TCS SP5 II confocal microscope (Leica Microsystems CMS GmbH, Mannheim, Germany) with a 63× 1.4 NA oil immersion objective in the inverted configuration. Quantitative analysis of colocalization was performed performed with NIS-Elements software (Nikon Instruments, Melville, NY, USA).

### Homogeneous time-resolved fluorescence cAMP assays

The cAMP quantification was performed performed with a homogeneous time-resolved fluorescence (HTRF) cAMP detection kit (cAMP HiRange; Cisbio, Bagnols/Cèze Cedex, France) as described previously 22. Human embryonic kidney 293 cells were dispensed with 25 μl of compound buffer consisting of minimal essential medium supplemented with 0.5 mM isobutylmethylxanthine (Sigma-Aldrich, St. Louis, MO, USA), 0.2% fatty acid-free bovine serum albumin (Sigma-Aldrich), 0.5 mg/ml zeocin, 0.5 mg/ml geneticin or 0.5 mg/ml hygromycin at 2–6 × 10^4^ cells/well in 96 half-well plates (Costar, Corning, NY, USA) on the day of the experiment. After an incubation of 1 hr at 37°C in a humidified 5% CO_2_ incubator, 25 μl of compound buffer containing 10 μM forskolin and desired concentrations of buprenorphine (Sigma-Aldrich) were added to the cells, followed by 30-min. incubation at room temperature. U-69593, DAMGO and nociceptin (Tocris Bioscience) — which are a KOP, a MOP and a NOP receptor agonist, respectively — were included as the positive control. To evaluate AC superactivation, desired concentrations of drugs were added to the compound buffer and incubated at 37°C for 4 hrs; the compound buffer was then replaced by 10 μM forskolin with 1 μM naloxone. Subsequently, 25 μl of cAMP-d2 and 25 μl of anti-cAMP cryptate conjugate were added to each well. After 1-hr incubation at room temperature, the plate was read on a FlexStation 3 microplate reader (Molecular Devices, Silicon Valley, CA, USA) with emission wavelength at 615 and 665 nm. The cAMP concentrations were calculated by non-linear regression analysis with SoftMax Pro (Molecular Devices, Sunnyvale, CA, USA). Concentration-response curves of cAMP accumulation, potency (pIC_50_) and efficacy (*E*_max_) for inhibition of forskolin-stimulated cAMP formation by U-69593, DAMGO, nociceptin and buprenorphine were analysed using Prism (GraphPad Software) 29,30.

### Statistical analysis

All results are expressed as the mean ± SE values of *n* experiments. One-way anova followed by Tukey's test was used to determine whether the difference is statistically significant (*P* < 0.05).

## Results

### Establishment of HEK 293 cells stably expressing FLAG-tagged KOP receptor

We have established an *in vitro* cell model by overexpressing FLAG-tagged human KOP along with HA-tagged MOP and myc-tagged NOP receptors in HEK 293 cells. Plasmids harbouring FLAG-tagged KOP receptor were transfected in HEK 293 cells expressing HA-tagged MOP or myc-tagged NOP receptors individually or simultaneously, and the stable clones were selected by appropriate antibiotics. AsAC activity is the major end-point measured in this study, the four stable clones presented here (KOP-, KOP+MOP-, KOP+NOP- and KOP+MOP+NOP-expressing cells) were chosen based on the strongest AC inhibition by acute treatment of 1 μM of U-50488, a selective KOP agonist, compared to other stable clones during an initial screening. Subsequent experiments demonstrated similar AC inhibition levels between the KOP-, KOP+MOP-, KOP+NOP- and KOP+MOP+NOP-expressing cells elicited by another KOP receptor agonist, U-69593 (Fig.3A and Table2). In saturation radioligand binding studies, [15,16-^3^H]-diprenorphine displayed a similar affinity (*K*_D_) for all stably transfected cell lines harbouring KOP receptors; but cells expressing KOP+NOP receptors had non-significantly less diprenorphine-binding sites than the other three transfected cell lines, as reflected by the lower *B*_max_ value (Table1).

**Table 1:** *B*_max_ (pmol/mg protein) and *K*_D_ (nM) values of KOP receptors expressed in HEK 293 cells coexpressing KOP, MOP and NOP receptors

Receptor(s)	[15,16-^3^H]-diprenorphine	
	*B*_max_ (pmol/mg protein)	*K*_D_ (nM)
KOP	2.827 ± 0.717	2.62 ± 0.48
KOP+MOP	2.509 ± 0.687	3.00 ± 0.77
KOP+NOP	1.574 ± 0.195	2.75 ± 0.26
KOP+MOP+NOP	2.512 ± 0.654	3.48 ± 0.69

Saturation binding assays were performed with [15,16-^3^H]-diprenorphine. Each value represents the mean ± SE of six to seven experiments performed in duplicate. No significant differences were found among the *B*_max_ and *K*_D_ values of these four stable clones.

**Table 2:** Potency (pIC_50_) and efficacy (*E*_max_) for inhibition of forskolin-stimulated cAMP formation by U-69593, DANGO, nociceptin and buprenorphine in HEK 293 cells expressing KOP, MOP and NOP receptors

Drug	Receptor(s)	pIC_50_	*E*_max_ (% inhibition)
U-69593	KOP	9.375 ± 0.179	90.67 ± 2.40*
	KOP+MOP	9.038 ± 0.178	82.78 ± 3.73
	KOP+NOP	9.206 ± 0.131	83.01 ± 2.61
	KOP+MOP+NOP	9.162 ± 0.278	84.92 ± 4.56
DAMGO	KOP	6.287 ± 0.571	85.76 ± 40.43
	KOP+MOP	7.305 ± 0.256	85.85 ± 8.44
	KOP+NOP	9.924 ± 2.306	29.39 ± 3.37*
	KOP+MOP+NOP	7.578 ± 0.217	88.64 ± 6.24
Nociceptin	KOP	8.548 ± 2.531	22.89 ± 7.11*
	KOP+MOP	7.954 ± 1.630	32.61 ± 10.82*
	KOP+NOP	10.50 ± 0.435	66.20 ± 3.11
	KOP+MOP+NOP	10.31 ± 0.175	91.55 ± 1.71
Buprenorphine	KOP	8.058 ± 0.302	69.93 ± 3.54
	KOP+MOP	8.055 ± 0.321	68.24 ± 3.29
	KOP+NOP	7.189 ± 0.397	69.61 ± 6.97
	KOP+MOP+NOP	6.886 ± 0.329	88.71 ± 6.84*

* Significant difference (*P* < 0.05) among the *E*_max_ values of the four stable clones treated with the same drug according to one-way anova with Tukey's multiple comparisons test.

Values represent the mean ± SE of five to fourteen experiments performed in duplicate as described in Figure3.

**Figure 1:**
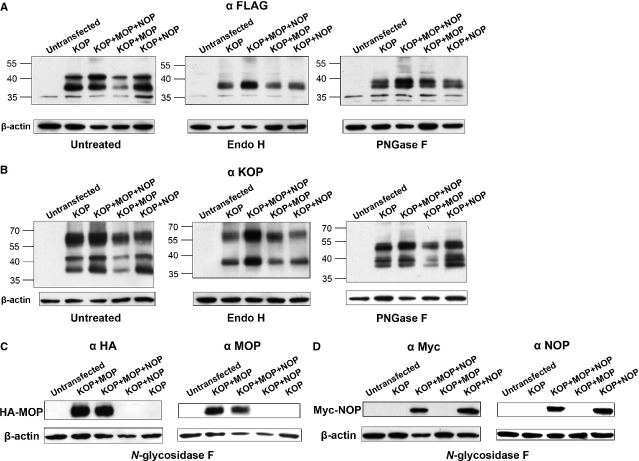
KOP, MOP and NOP receptors expressed in HEK 293 cells are *N*-linked glycoproteins. Cell lysates were prepared by extracting HEK 293 cells expressing FLAG-KOP, HA-MOP or myc-NOP receptors in lysis buffer for 1 hr on ice. Cellular debris was pelleted by centrifugation; the supernatants were treated with Endo H, PNGase F (KOP) (**A** and **B**), or *N*-glycosidase F (MOP and NOP) (**C** and **D**) (protease-free, 50 units/mg of membrane protein) at 37°C for 3 hrs, and then resolved using 10% SDS-PAGE. Receptors were detected by immunoblotting using the polyclonal rabbit anti-FLAG, anti-KOP, anti-MOP, anti-NOP, monoclonal mouse anti-HA or anti-myc antibody.

Western blot analysis using anti-FLAG and anti-KOP receptor antibodies revealed that the introduced human KOP receptor was expressed as three major heterogeneous forms in HEK 293 cells, with apparent molecular masses of ~37, 43 and 60 kD (Fig.1A and B). The predicted molecular mass for the FLAG-tagged human KOP receptor is 43.7 kD. As the apparent molecular weights of the overexpressed KOP receptor were different from the expected values, we suggested that the overexpressed human KOP receptor might undergo glycosylation 31. Evidence that the human KOP receptor is a glycoprotein was provided by digestion with Endo H, a recombinant glycosidase which cleaves within the chitobiose core of high mannose and some hybrid oligosaccharides from *N*-linked glycoproteins, and PNGase F, an amidase that cleaves between the innermost GlcNAc and asparagine residues of high mannose, hybrid, and complex oligosaccharides from *N*-linked glycoproteins. Endo H and PNGase F treatment of the KOP receptor increased the mobility of the 43 and 60 kD bands to species of apparent molecular masses of 37 and 39 (and 50) kD respectively. The reason for the aberrant electrophoretic mobility of the receptor, even after deglycosylation, is unknown at present 32. Immunoblotting of the *N*-glycosidase F-treated MOP and NOP receptors was also performed to confirm the coexpression of the previously introduced MOP or NOP receptor and the newly introduced KOP receptor in the HEK 293 cells (Fig.1C and D).

**Figure 2:**
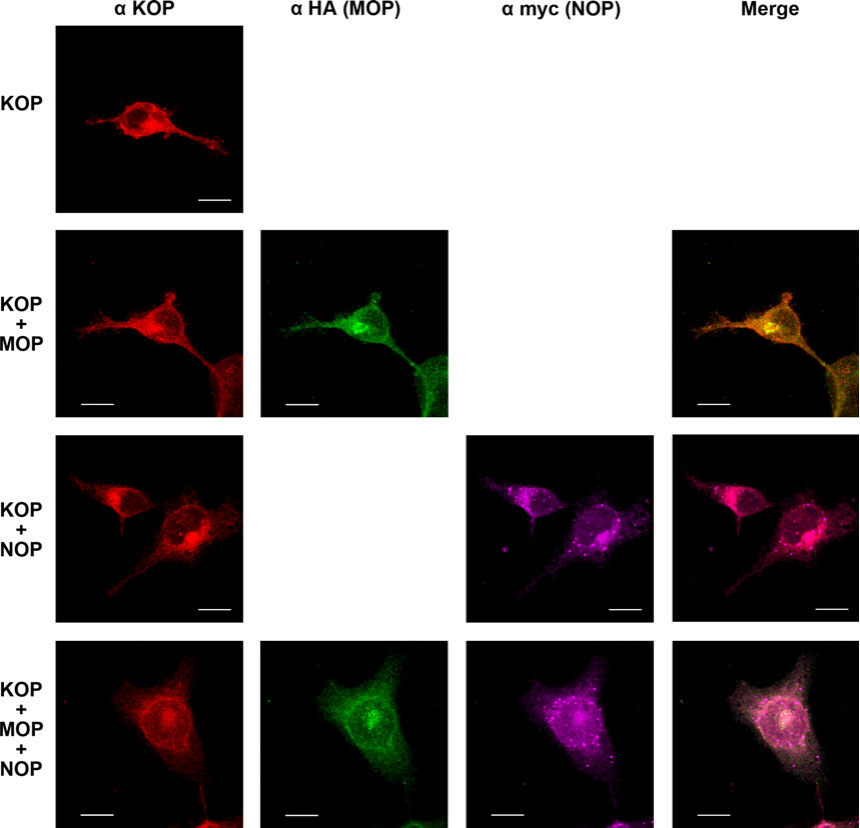
Representative confocal images from HEK 293 cells expressing KOP, KOP+MOP, KOP+NOP and KOP+MOP+NOP receptors. FLAG-tagged KOP receptor was detected with anti-KOP rabbit polyclonal antibody and visualized by Alexa Flour 594 goat anti-rabbit antibody (red); HA-tagged MOP receptor was detected with anti-HA mouse monoclonal antibody and visualized by Alexa Flour 488 goat antimouse antibody (green); myc-tagged NOP receptor was detected with anti-myc chicken IgY fraction and visualized by Alexa Flour 647 donkey anti-chicken IgY antibody (magenta). The colocalization of KOP, MOP and NOP receptors is depicted in the merged pictures. Scale bars are equal to 10 μm.

We next examined whether the KOP receptor colocalizes with coexpressed MOP or NOP receptor. Shown in Figure2 are confocal fluorescence images of HEK 293 cells expressing FLAG-tagged KOP, HA-tagged MOP and myc-tagged NOP receptors. The KOP receptor was clearly present in the vesicles distributed throughout the cytoplasm and also on the plasma membrane. The MOP and NOP receptors localizes to the cell surface as well as vesicular structures, and prominently colocalizes with KOP receptors (Mander's overlap: 87.5% for KOP and MOP receptors, 89.5% for KOP and NOP receptors and 82.7% for MOP and NOP receptors).

### AC inhibition after acute opioid exposure

**Figure 3:**
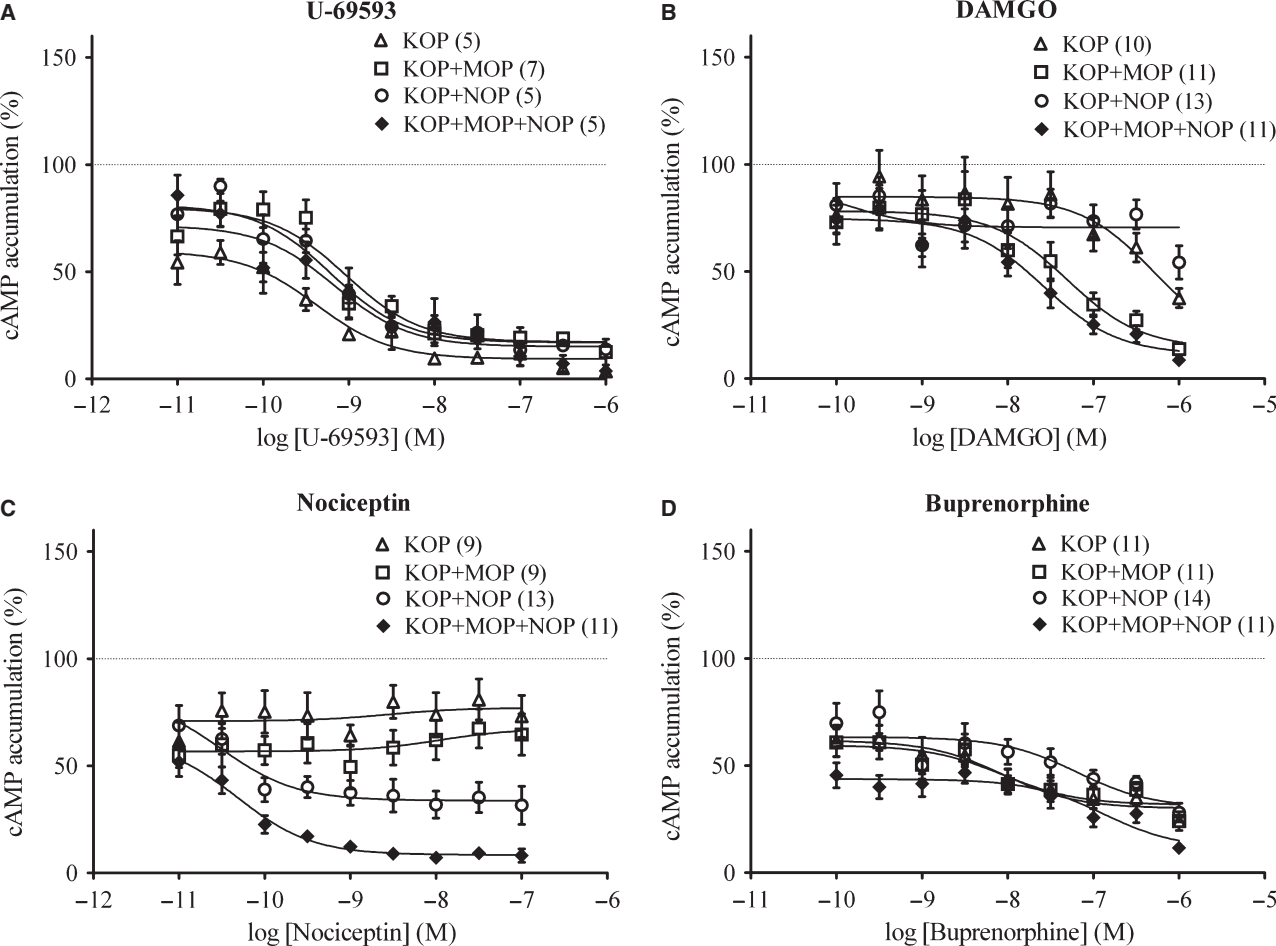
Effects of acute exposure to U-69593, DAMGO, nociceptin or buprenorphine on forskolin-stimulated cAMP accumulation in HEK 293 cells expressing KOP, KOP+MOP, KOP+NOP and KOP+MOP+NOP receptors. HEK 293 cells expressing KOP (*open triangle*), KOP+MOP (*open square*), KOP+NOP (*open circle*) or KOP+MOP+NOP (*filled diamond*) were treated with U-69593 (**A**), DAMGO (**B**), nociceptin (**C**) or buprenorphine (**D**) for 30 min. at room temperature in the presence of 10 μM forskolin prior to HTRF cAMP assays. Each point represents the mean ± SE value of five to fourteen (numbers indicated in the parentheses) experiments performed in duplicate using different batches of cells. 100% defines forskolin-stimulated cAMP accumulation in cells not treated with aforementioned drugs.

Effects of acute exposure to U-69593, DAMGO, nociceptin and buprenorphine on KOP-, MOP- or NOP-mediated G_i/o_-coupled AC inhibition were examined. U-69593 concentration dependently inhibited forskolin-stimulated cAMP accumulation in all HEK 293 cell lines expressing KOP receptors (Fig.3A; Table2). In HEK 293 cells not expressing MOP receptor, DAMGO did not inhibit forskolin-stimulated AC activity at concentrations lower than 0.1 μM (Fig.3B, open triangles and circles). On the other hand, in HEK 293 cells expressing no NOP receptor, nociceptin did not inhibit forskolin-stimulated AC activity at concentrations lower than 0.1 μM (Fig.3C, open triangles and squares). These results suggest that the specific KOP, MOP and NOP agonists specifically act on the introduced KOP, MOP and NOP receptors respectively.

Buprenorphine displayed a relatively flat concentration-inhibition curve on cAMP accumulation in KOP-and KOP+MOP-expressing cells with efficacy of ~70% inhibition (Fig.3D, open triangles and squares; Table2), while the efficacy of the known full KOP agonist U-69593 is ~85–90% (Fig.3A, open triangles and squares; Table2); supporting its KOP receptor partial agonist and MOP receptor partial agonist characteristics. In NOP-expressing cells, buprenorphine at higher concentrations (>30 nM) inhibited cAMP accumulation in a concentration-dependent manner (Fig.3D, open circles and filled diamonds; Table2), resembling nociceptin (Fig.3C, open circles and filled diamonds; Table2), a NOP receptor agonist. This suggests that buprenorphine acts as an NOP receptor agonist at higher concentrations in the presence of coexpressed KOP receptor. Interestingly, buprenorphine showed a lower potency (lower pIC_50_) (Table2) but higher efficacy (higher *E*_max_; Table2) in KOP+MOP+NOP-coexpressing cells than in cells expressing KOP+NOP receptors (Fig.3D; Table2). Similarly, the AC inhibition curves elicited by nociceptin showed higher efficacy (higher *E*_max_) in KOP+MOP+NOP- than KOP+NOP-expressing cells (Fig.3C; Table2). This suggests that the effect of buprenorphine on KOP+MOP+NOP-expressing cells is not solely owing to its agonistic property on NOP receptor, and might be enhanced by coexpressed MOP receptor.

### AC superactivation after chronic opioid treatment

**Figure 4:**
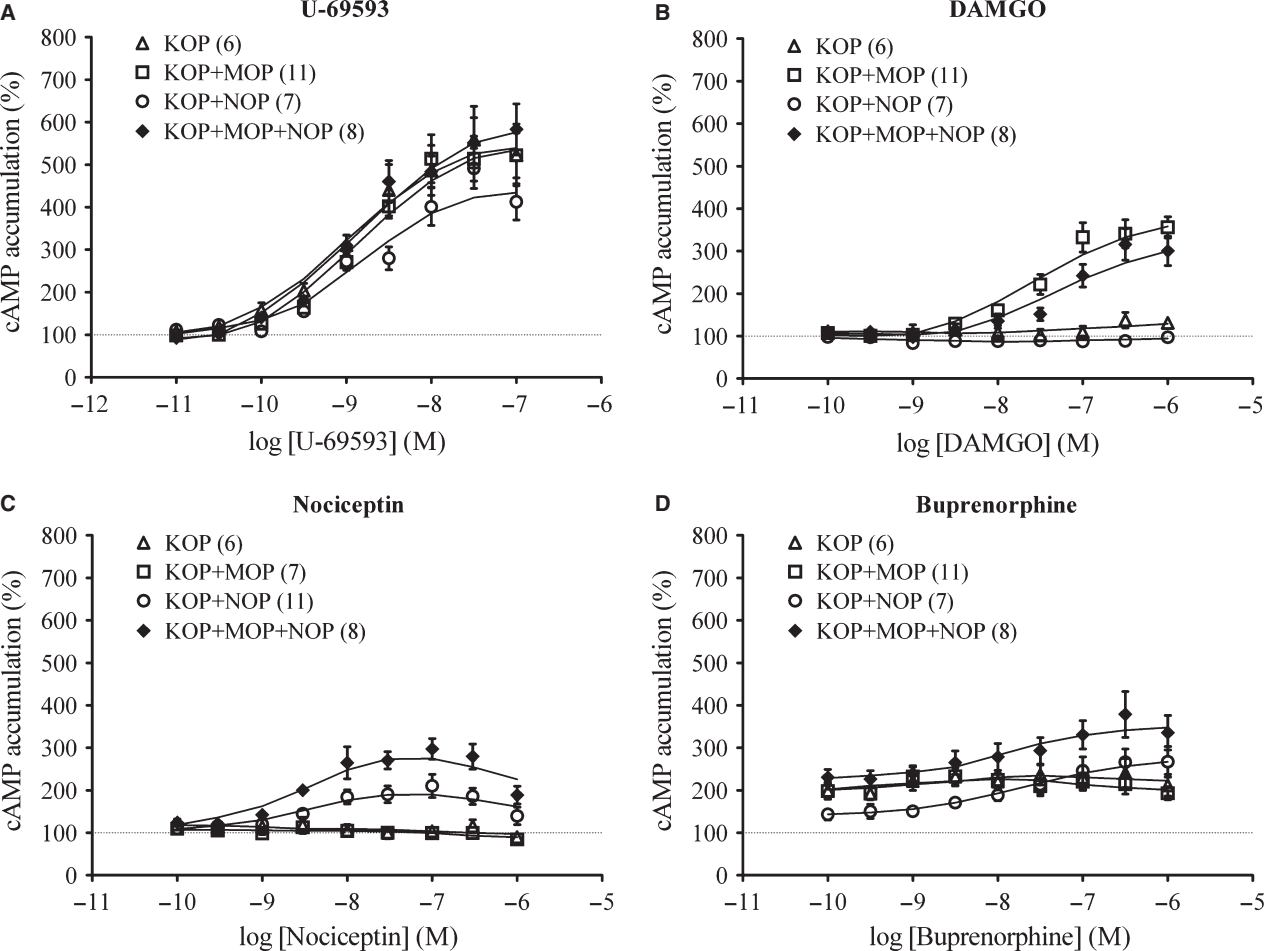
Naloxone precipitation on the effects of chronic exposure to U-69593, DAMGO, nociceptin or buprenorphine on forskolin-induced cAMP accumulation in HEK 293 cells expressing KOP, KOP+MOP, KOP+NOP and KOP+MOP+NOP receptors. After exposure to U-69593 (**A**), DAMGO (**B**), nociceptin (**C**) or buprenorphine (**D**) for 4 hrs at 37°C, the incubation media were subsequently removed, and HEK 293 cells expressing KOP (*open triangle*), KOP+MOP (*open square*), KOP+NOP (*open circle*) or KOP+MOP+NOP (*filled diamond*) were treated with 1 μM naloxone accompanied by 10 μM forskolin immediately prior to HTRF cAMP assays. Each point represents the mean ± SE value of six to eleven (numbers indicated in the parentheses) experiments performed in duplicate using different batches of cells. 100% defines forskolin-stimulated cAMP accumulation in cells treated with none of the aforementioned drugs.

Next, we extended our investigation by adding naloxone to cells exposed to drugs for 4 hrs, an incubation period reported to show a prominent overshoot in forskolin-stimulated cAMP accumulation 33. When HEK 293 cells expressing KOP, KOP+MOP, KOP+NOP and KOP+MOP+NOP receptors were exposed to U-69593 for 4 hrs, addition of naloxone revealed AC superactivation, as shown by the overshoot (up to 550%) of cAMP accumulation (Fig.4A). No AC superactivation was observed in KOP- and KOP+NOP-expressing cells chronically exposed to DAMGO, whereas KOP+MOP-expressing cells displayed slightly higher AC superactivation than KOP+MOP+NOP-expressing cells (Fig.4B). KOP+MOP+NOP-expressing cells displayed greater AC superactivation than KOP+NOP-expressing cells after chronic exposure to nociceptin with bell-shaped concentration-response curves, while KOP- and KOP+MOP-expressing cells exhibited no AC superactivation (Fig.4C). Interestingly, AC superactivation did occur in KOP+NOP- and KOP+MOP+NOP-expressing cells chronically exposed to buprenorphine, and KOP+MOP+NOP-expressing cells even exhibited larger magnitude of AC superactivation than KOP+NOP-expressing cells (Fig.4D).

## Discussion

### Crosstalk of KOP, MOP, and NOP receptors

We demonstrated the colocalization of coexpressed human KOP, MOP and NOP receptors in HEK 293 cells (Fig.2). The high colocalization rate suggests the close proximity of the KOP, MOP and NOP receptors, or even formation of heterodimerized MOP-NOP, MOP-KOP or KOP-NOP receptors 26,34. Our saturation binding assay using [15,16-^3^H]-diprenorphine (Table1) — which showed that coexpressing KOP with MOP and NOP receptors non-significantly decreased the diprenorphine affinity of the receptor (higher *K*_D_) — also implies the novel properties of coexpressed human KOP+MOP+NOP receptors. In competition binding assays, the binding potencies (K_i_) of diprenorphine for mouse KOP (U-69593) and rat MOP (DAMGO) receptors are 0.017 nM and 0.072 nM, respectively 35, whereas diprenorphine is inactive (K_i_ > 10 μM) in binding human NOP (nociceptin) receptors 36. Therefore, in clones expressing both KOP and MOP receptors, the *K*_D_ and *B*_max_ values reported might represent a mixture of diprenorphine binding to KOP, MOP and presumably KOP-MOP heterodimeric receptors. Interestingly, the receptor density (*B*_max_) of the cells expressing solely KOP receptors was similar to those of the cells expressing KOP+MOP and KOP+MOP+NOP receptors, but non-significantly higher than that of KOP+NOP-expressing cells. The coexpressed MOP receptors did not result in an increase, while the coexpressed NOP receptors resulted in a non-significant decrease in the *B*_max_ reported for diprenorphine in these clones is intriguing. As the expression levels of FLAG-tagged KOP receptors are not drastically different in cells expressing KOP receptor alone and coexpressing MOP or NOP receptors as revealed by immunoblotting (Fig.1), the variation in *B*_max_ (Table1) might be because of the different numbers of KOP receptor transported from ER-Golgi to the plasma membrane 37. Another possibility is that coexpressed human KOP, MOP and NOP receptors, perhaps forming heteromers, adopted a different conformation of the diprenorphine-binding site from homomeric KOP and MOP receptors, rendering the binding affinity non-significantly lower than KOP receptor homomers (Table1, *K*_D_ values).

### Acute activation of KOP receptors inhibits AC activity

Acute agonist exposure inhibits forskolin-induced accumulation of cAMP in recombinant HEK 293 cells expressing cloned opioid receptors; this effect is mediated by the inhibition of AC activity upon opioid receptor activation 38,39. In our cell model, acute treatment with the KOP receptor agonist, U-69593, specifically repressed the AC activity in cells expressing recombinant KOP receptor (Fig.3A). The MOP and NOP receptor agonists, DAMGO and nociceptin, acutely inhibited AC in cells coexpressing KOP with MOP and NOP receptors respectively (Fig.3B and C). Previous study showed that buprenorphine acts as both a MOP and KOP receptor agonist on mouse vas deferens 40. Buprenorphine, which acted as a partial agonist instead of an antagonist at KOP (Fig.3D), a partial agonist at MOP and a full agonist at NOP receptors, exhibited the highest efficacy (*E*_max_) in cells coexpressing KOP, MOP and NOP receptors in comparison to the other three transfected cell lines (Table2). This highest response might result from the hetero-oligomerization of KOP, MOP and NOP receptors,or the simultaneous regulation of common secondary messengers by KOP, MOP and NOP receptors.

### AC superactivation induced by buprenorphine and nociceptin

The 4-hrs buprenorphine exposure induced prominent forskolin-induced cAMP accumulation (~3.5-fold) in KOP+MOP+NOP-expressing cells and relatively low cAMP accumulation (~2-fold) in the other three KOP-expressing cell lines (Fig.4D). Another interesting observation is the bell-shaped curve of nociceptin-induced AC superactivation (Fig.4C), which is reminiscent of our previous observation for Ro 64-6198, a non-peptide NOP receptor agonist 22. This biphasic response might reflect an intrinsic NOP receptor property that receptor activation by higher concentration (above 0.1 μM) of agonists would generate a reduction, not an enhancement, of AC superactivation, thus contributing to its role in modulating opioid anti-nociception 41 and blocking the rewarding effects of several abused drugs, including morphine 42, cocaine 43 and amphetamine 44. The difference between buprenorphine and nociceptin remains to be elucidated if it is because of the difference between the partial agonist (buprenorphine) and full agonist (nociceptin) for NOP receptors, or owing to the action of buprenorphine on KOP and MOP receptors that expressed simultaneously.

### Clinical implications

When the responses to long-term treatment of DAMGO and buprenorphine are compared, cells expressing KOP, MOP and NOP receptors display matching concentration-response curves at the sub-μM range (Fig.4B and D, filled diamonds). In contrast, cells expressing KOP+MOP concentration dependently responded to DAMGO, not to buprenorphine (Fig.4B and D, open squares), whereas KOP+NOP-expressing cells exhibited concentration-dependent response to buprenorphine but not to DAMGO (Fig.4B and D, open circles). This implies that chronic DAMGO and buprenorphine treatments induce differential effects on cells expressing either KOP+MOP or KOP+NOP, yet lead to similar cellular responses in the context of coexpressed KOP+MOP+NOP receptors at the sub-μM concentrations. More importantly, buprenorphine elicited a drastically high cAMP accumulation (~6-fold) in cells expressing NOP alone in our previous study 22, and this change was attenuated by coexpressed KOP receptors (Fig.4D, open circles). In the recent years, significant attention has elaborated the non-CNS role of MOP and NOP receptors. The MOP receptor has been implicated in the angiogenesis and cancer progression when used on a chronic basis 45,46. Similarly, NOP receptor has been implicated in renal function along with MOP and KOP receptors 47–49. All these functions are associated with AC superactivation and under chronic conditions. As the cells used are also from the kidney (HEK), not the CNS, these data may have relevance to the roles of MOP/NOP receptors in cancer and renal pathology. Therefore, our cellular model could mimic the physiological responses of specific cells that coexpressing KOP, MOP and NOP receptors in patients under buprenorphine maintenance therapy, afflicted by cancer, or with kidney diseases. KOP receptor is known to be associated with stress-induced craving and relapse risk 50; hence, our cellular model might be applied to investigating the relationship between the KOP receptor and the relapse of illicit drug use in the future.

In summary, our study showed that buprenorphine could inhibit AC activity almost completely in cells expressing KOP+MOP+NOP receptors when applied acutely; after chronic exposure to buprenorphine, AC superactivation evoked *via* KOP and NOP receptors could be enhanced by coexpressing MOP receptor, and NOP receptor is crucial for the concentration-dependent AC superactivation. The *in vitro* cell model of coexpressed human KOP+MOP+NOP receptors provides insight into cross-talk among opioid receptors following opioid exposure, and could provide a new platform to screen novel drugs for opioid addiction or analgesic therapy.
